# Complete Vesicourethral Anastomotic Disruption Following Prostatectomy

**DOI:** 10.1089/cren.2016.0065

**Published:** 2016-06-01

**Authors:** Ashima Singal, Christopher M. Gonzalez, Daniel Oberlin, Justin S. Han

**Affiliations:** ^1^ Department of Urology, Feinberg School of Medicine, Northwestern University, Chicago, Illinois.; ^2^ Department of Urology, Case Western Reserve University, Cleveland, Ohio.; ^3^ Department of Urology, North Shore University Hospital, New Hyde Park, New York.

## Abstract

Vesicourethral anastomotic (VUA) disruption with bladder displacement into the abdominal cavity following robot-assisted laparoscopic prostatectomy (RALP) is an exceedingly rare complication. There have been no cited case reports after robotic surgery but case reports after open radical prostatectomy have been noted. Other complications related to VUA include bleeding with or without pelvic hematoma, bladder neck contracture, or severe stress urinary incontinence. Following radical prostatectomy, studies estimate the rate of VUA leakage to be 1.4% and no exact rate of complete disruption is known given its rarity. However, the majority of these cases are managed conservatively and rarely require reoperation. To date, there are no published studies that describe complete VUA and bladder displacement secondary to a large pelvic hematoma following prostatectomy. We report a rare case of VUA disruption after RALP successfully managed with conservative treatment.

## Clinical History

A 63-year-old male with history of Gleason 7 adenocarcinoma of the prostate, hyperlipidemia, and hypertension was admitted for complications following a robot-assisted laparoscopic prostatectomy (RALP) at an outside hospital. His initial operation was complicated by hemorrhage requiring transfusion of 10 U of packed red blood cells in the intra- and peri-operative periods. On postoperative day 15, he had one readmission for endoscopic clot evacuation procedures at the outside hospital for persistent postoperative hemorrhage and a suprapubic catheter (SPC) was placed for maximal urinary drainage. He was discharged and returned on postoperative day 27 presenting with altered mental status, found to be septic and in clot retention. He underwent an exploratory laparotomy where 4 L of clot was evacuated via cystotomy along with a large abdominal hematoma. In addition to the SPC and foley, two drains were placed in the pelvis. Due to an incomplete response to transfusion and persistent hemorrhage, as well as concern for small bowel evisceration, the patient was transferred to our tertiary care medical center for further management on postoperative day 40.

## Physical Exam

When presenting to our institution vitals were as follows: temperature 98.6, blood pressure 126/72, heart rate 117, oxygen saturation 99% on 2 L nasal canula. The patient was found to be in no acute distress with naso-gastric tube in place. The abdomen was distended with no rebound or guarding. There was a right and left lower quadrant drains in place and midline incision with retention sutures in place. The lower aspect of the incision was inspected and found to have loops of bowel visible. Patient had a three-way urethral catheter and a SPC, both 24F.

## Diagnosis

**FIG. 1.**
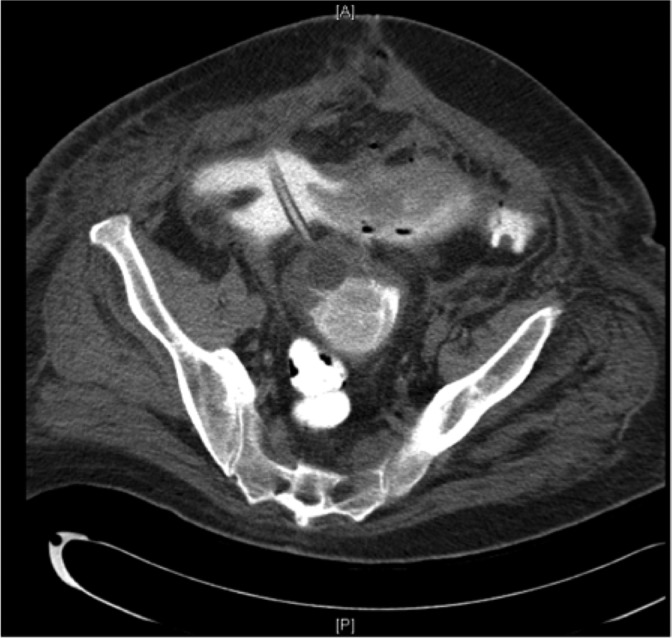
CT axial abdomen/pelvis with contrast. Extravasation of contrast from bladder. Large pelvic hematoma in close proximity to bladder. CT, computed tomography.

**FIG. 2.**
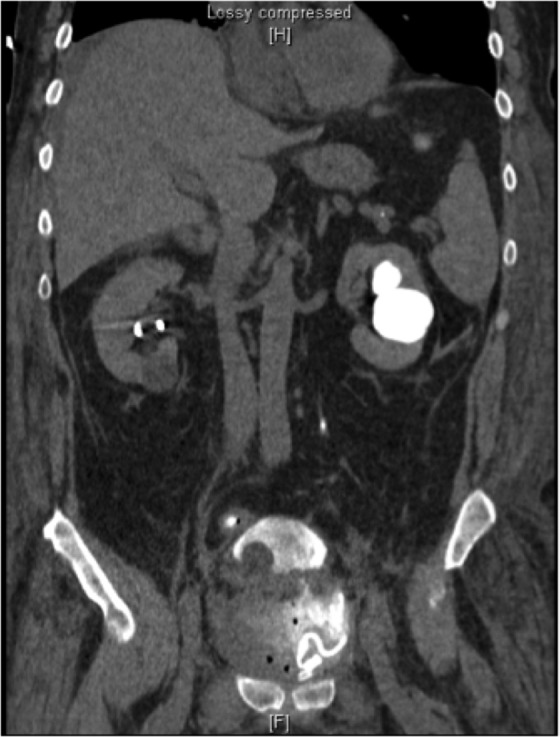
CT saggital abdomen/pelvis with contrast. Disruption of vesicourethral anastomosis with bladder displaced superiorly.

Initial imaging revealed vesicourethral anastomotic (VUA) disruption and extravasation of contrast into the intrapertioneal space (Figs. 1 and 2). Due to an incomplete response to transfusion and persistent hemorrhage, the patient was diagnosed with postsurgical bleeding and formation of pelvic hematoma. Regarding incision with visible bowel, general surgery was consulted and diagnosed the patient with wound dehiscence without evisceration (recommended conservative management rather than primary closure).

## Intervention

To manage postsurgical bleeding, interventional radiology (IR) was consulted and the patient underwent subsequent angioembolization of the anterior branch of the internal iliac artery. His hemoglobin subsequently stabilized. To manage VUA, IR concurrently placed bilateral percutaneous nephrostomy (PCN) tubes. Given the degree of VUA, retrograde bilateral stents would likely be quite difficult to place and were therefore deferred. Pelvic drains were removed on POD44. Patient was discharged and underwent several periodic endoscopic evaluations that consisted of cystoscopy and cystogram. Bilateral PCNs removed on postoperative day 90.

**FIG. 3.**
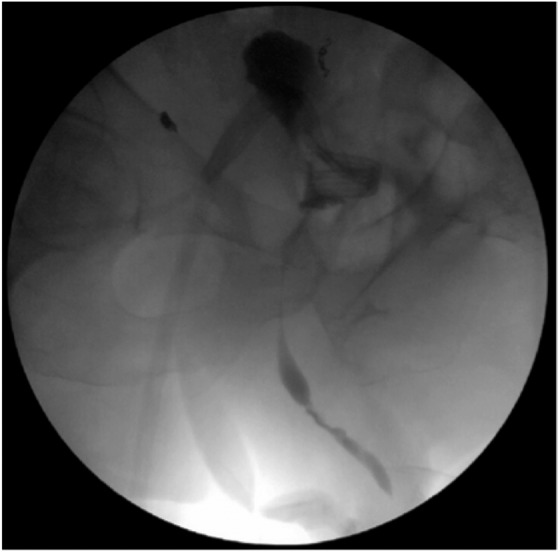
Intraoperative cystogram. Bladder high-riding in pelvis.

Six months postoperatively the patient underwent routine exchange of the urethral foley catheter and SPC, in addition to retrograde urethrogram and cystoscopy. Intraoperative cystogram showed a displaced bladder (Fig. 3). Serial cystograms over the next several months revealed hematoma resolution and continued caudal bladder migration into the pelvis.

**FIG. 4.**
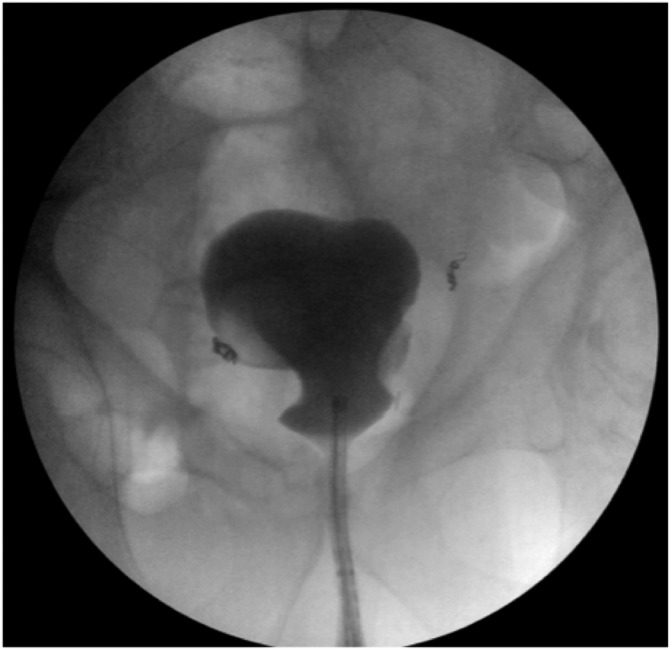
Intraoperative cystogram. Patent Urethra with continuity to bladder. Bladder in essentially normal anatomical position.

Foley was removed on postoperative month 7. With only a SPC remaining, a repeat study in postoperative month 8 demonstrated patency and continuity of the bladder neck to urethra (Fig. 4). The SPC was capped and the patient passed voiding trial, though he noted severe stress urinary incontinence (SUI) for several months postprocedure. Patient's SPC was removed on postoperative month 9 and he was started on kegel exercises for SUI.

## Follow-Up/Outcome

His SUI had improved with pelvic physical therapy from greater than five pads per day to only one pad per day as of December 2015. His prostate specific antigen remains undetectable.

## Discussion

Complete VUA disruption is a very rare complication that can be successfully managed with conservative treatment.^1^ Our proposed pathway for our patient's VUA disruption was secondary to a postoperative hematoma following RALP. During RALP surgery, the peritoneum and retroperitoneum are violated and a potential postoperative hematoma will no longer be confined to the retroperitoneum. A large hematoma, pushing against a mobilized bladder, may lead to the bladder becoming displaced into the abdominal space. This is the first case report describing this phenomenon.

We agree with the surgeon's initial decision for immediate exploration for bleeding and/or to prevent hematoma formation. Unfortunately, this patient had persistent bleeding that required additional intervention. Rarely, however, do patients require exploration^2^ or angioembolization for persistent bleeding. Common bleeding sites may include dorsal venous complex, accessory pudendal arteries, prostate pedicles, neurovascular bundles, external iliac vessels, and pelvic sidewall.

This patient presented with a pelvic hematoma and VUA disruption already formed. Given the rarity of VUA disruptions,^3^ and lack of guidelines or studies on how to manage this complication, it is unclear whether conservative therapy or operative intervention is preferred and therefore presents a challenging case to the urologist. Urgent exploration could resolve the hematoma and VUA disruption or remove the tension that kept the hematoma stabilized and cause additional rebleeding. Since the immediate period had passed, we felt there was little benefit to exploration. In delayed scenarios where a hematoma is already formed, we believe conservative management is effective. Our preferred strategy for management is maximal urinary diversion with bilateral PCNs, SPC, foley, and drains. This is followed by sequential endoscopic evaluation and individual removal to allow patient to heal, which can be a lengthy process (9 months in our patient).

In conclusion, our experience with this patient demonstrated that continued monitoring and patience for the patient and the surgeon is essential. We suggest that reconstructive intervention should be delayed until the hematoma is completely resolved and the bladder returns to the most dependent position possible. This avoids additional surgical complications and may preserve the continence of a patient. In this case, reconstructive surgery was entirely avoided and continuity between the bladder and urethra was progressively established.

## Abbreviations Used

CT: computed tomography
IR: interventional radiology
PCN: percutaneous nephrostomy
RALP: robot-assisted laparoscopic prostatectomy
SPC: suprapubic catheter
SUI: stress urinary incontinence
VUA: Vesicourethral anastomotic
